# Supplementary material for: Tyrosine kinase inhibitors affect sweet taste and dysregulate fate selection of specific taste bud cell subtypes via KIT inhibition
Source: Development. 2026 Apr 23;153(8):dev205259. doi: 10.1242/dev.205259 (PMC13143203; doi:10.1242/dev.205259)
Supplement: Supplementary information [file develop-153-205259-s1.pdf]

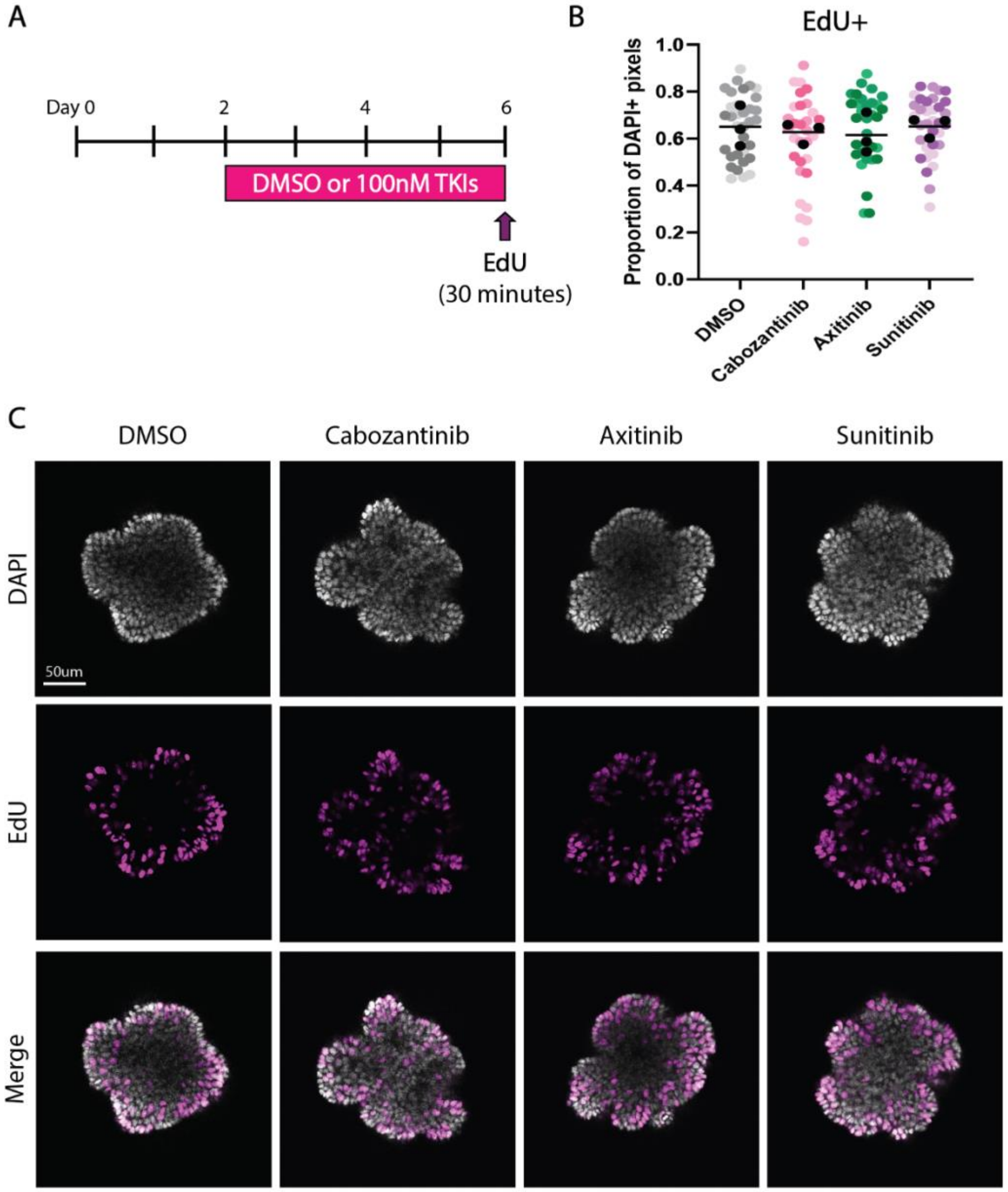

Fig. S1. TKIs do not affect proliferation of taste progenitor cells in organoids. (**A**) Timeline of lingual organoid treatment with DMSO or 100 nM TKIs during the growth phase (day 2-6). EdU was added to media for 30 minutes prior to harvest on day 6. (**B**) Drug treatment did not affect EdU labeling of organoids. Each colored dot represents the proportion of DAPI$^+$ pixels labeled with EdU in a single organoid. Within a treatment, different shades represent the 3 biological replicates, and black dots represent averages of each replicate. Ordinary one-way ANOVA with Dunnett's multiple comparisons test was used to compare the average values (black line) across conditions.

Development • Supplementary information

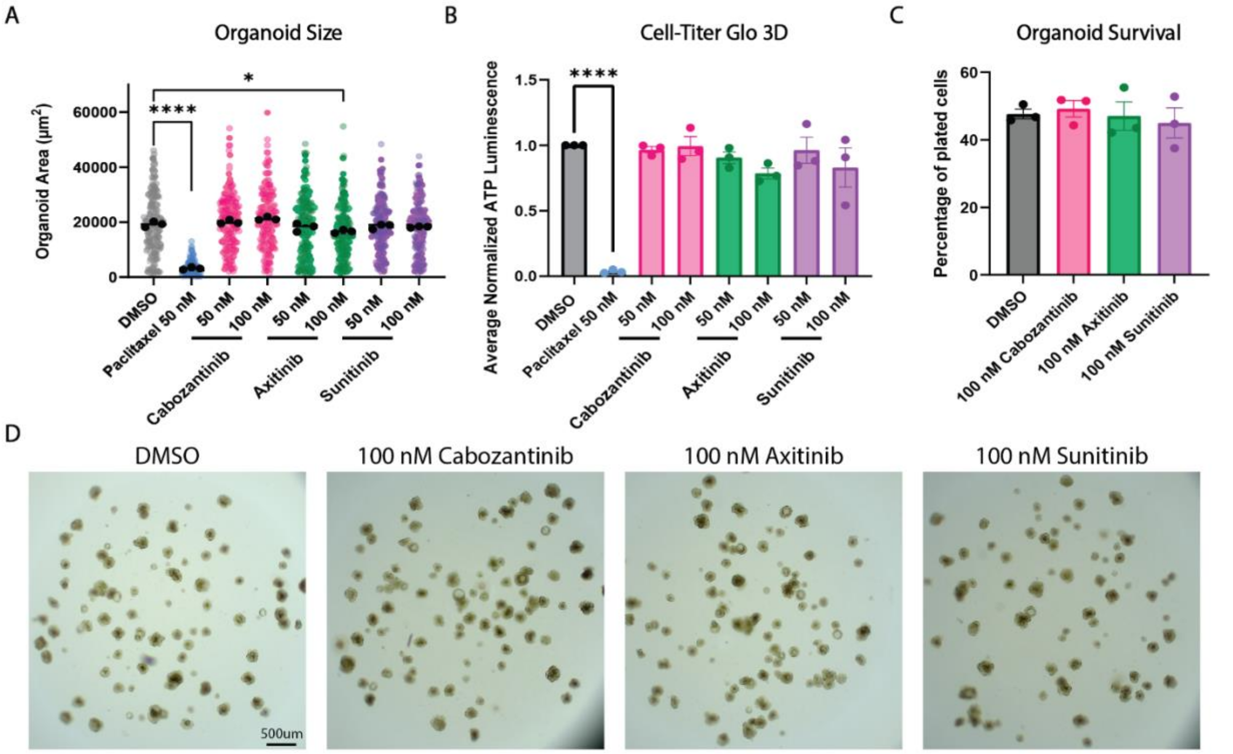

Fig. S2. TKI treatment during the growth phase of culture does not affect growth or survival of lingual organoids. (**A**) Organoid area was not broadly affected by treatment with 50 or 100 nM TKIs (see Fig 1A) compared to negative control (DMSO), but was significantly reduced by paclitaxel (positive control). Each colored dot represents the area of one organoid. Within a treatment condition, different shades represent the 3 biological replicates, and black dots represent averages of each replicate. (**B**) Estimation of cell survival using Cell-Titer Glo® 3D was unaltered by TKI treatment compared to DMSO control, but was significantly diminished by paclitaxel. Each dot represents the average luminescence across 6 pooled wells for each of 3 biological replicates. (**C**) Organoid survival did not differ with condition. The number of organoids in each well at day 6 was divided by 200 cells/well plated at day 0. Each dot represents the average of three wells for each of 3 biological replicates. Ordinary one-way ANOVA with Dunnett's multiple comparisons test was performed on experimental averages in panel A and on all values in panels B and C. Mean +/- SEM (* $p \leq 0.05$, **** $p \leq 0.0001$). (**D**) Brightfield images of culture wells containing organoids treated with DMSO or 100 nM TKIs.

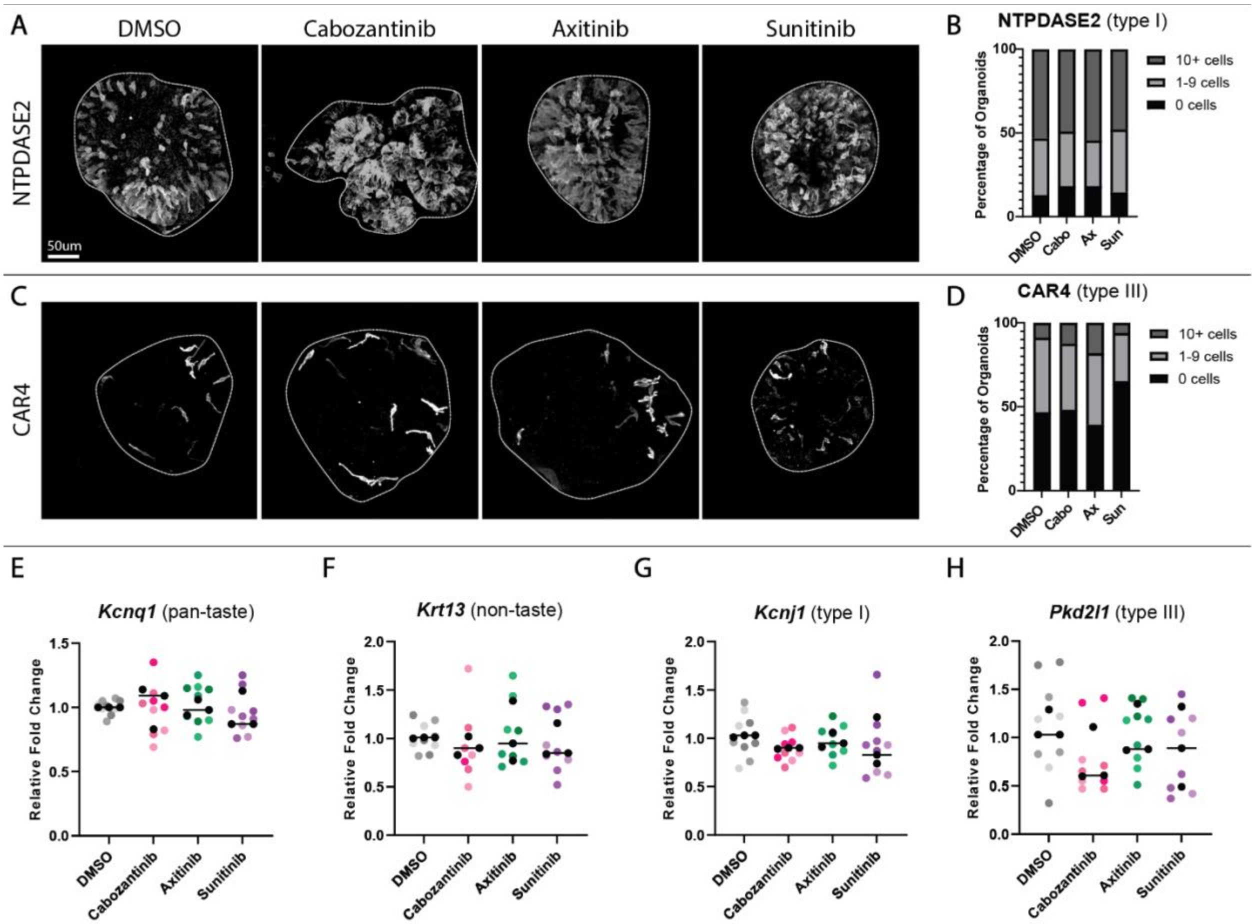

Fig. S3. TKIs do not affect type I or type III TBCs or markers of taste vs non-taste epithelium. Compressed confocal z-stacks of control and TKI-treated organoids immunostained for markers of type I (NTPDase2) (**A**) and type III (CAR4) TBCs (**C**). Histograms of percentages of organoids containing 0, 1-9, or ≥ 10 cells immunopositive for NTPDase2 (**B**) or CAR4 (**D**) reveal no change with drug treatment compared to controls. Total organoids obtained from 3 biological replicates: DMSO - 47; cabozantinib - 55; axitinib - 33; sunitinib - 48. Relative fold change in expression of pan-taste marker *Kcnq1* (**E**), non-taste marker *Krt13* (**F**), type I TBC marker *Kcnj1* (**G**) and type III TBC marker *Pkd2l1* (**H**) measured via RT-qPCR. Each colored dot represents an individual sample where organoids were pooled from three culture wells (~240 organoids per sample). Within a treatment, different shades represent the 3 biological replicates, and black dots represent averages of each replicate. Ordinary one-way ANOVA with Dunnett's multiple comparisons test was used to compare the average values (black line) for each marker across conditions.

Development • Supplementary information

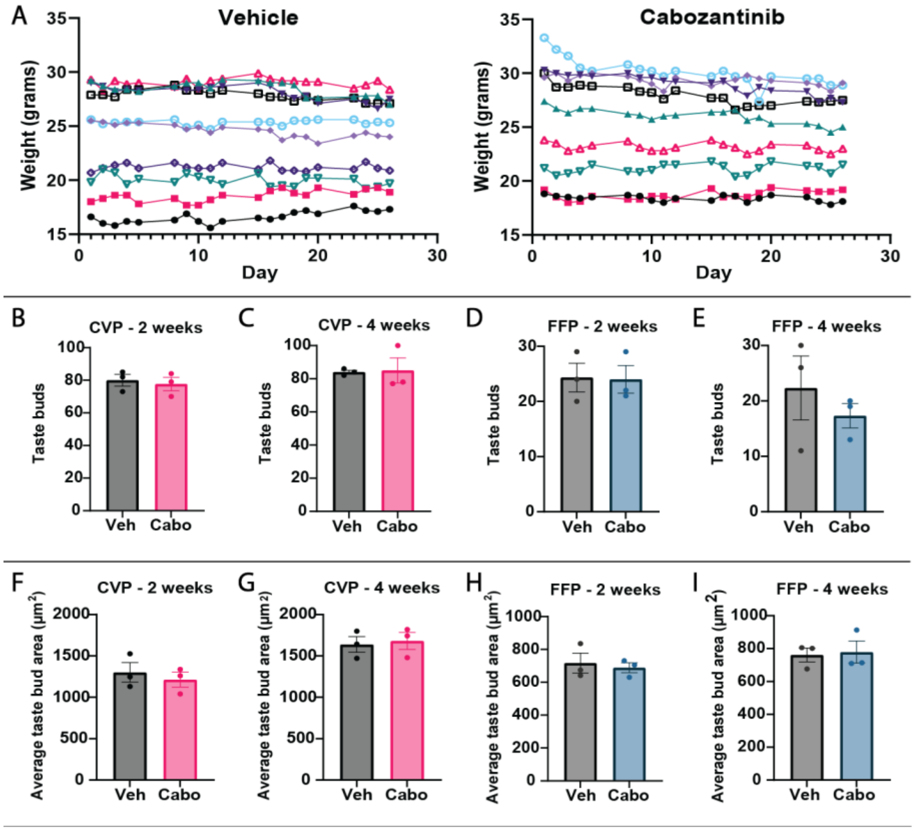

Fig. S4. Cabozantinib does not affect mouse health or general taste bud homeostasis. (**A**) Weights of mice treated with vehicle or cabozantinib did not differ over 28 days. Weight was measured 5 days per week (symbols) and each colored line is an individual mouse (N=9 mice per condition). The number of CVP taste buds did not differ with treatment at 2 weeks (**B**) or 4 weeks (**C**). The number of FFP taste buds in the first 1.2 mm of the tongue was unaltered by cabozantinib treatment at 2 weeks (**D**) or 4 weeks (**E**). The average area of CVP (**F-G**) and FFP (**H- I**) taste bud profiles did not differ with treatment at 2 weeks or 4 weeks (N=3 mice per condition). Unpaired t-test. Mean +/- SEM.

Development • Supplementary information

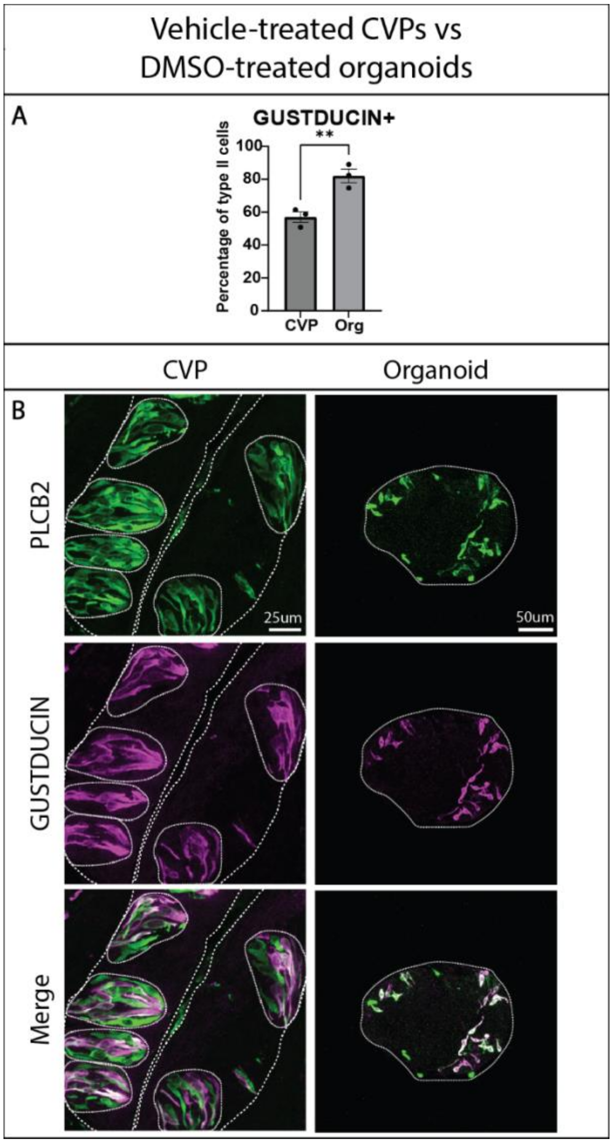

Fig. S5. GUSTDUCIN$^+$ type II cells are over-represented in lingual organoids compared to CVP taste buds. (A) The percentage of PLCβ2$^+$ type II cells expressing GUST is higher in organoids compared to *in vivo*. CVP values are from vehicle-treated mice after 4 weeks (see Figure 2; note that images are also reproduced from Fig. 2H,N), while organoid values are from organoids treated with DMSO during the differentiation phase (see **Figure 1**). Each dot in the organoid condition is the average percentage across ~10 organoids for each biological replicate. (**B**) Compressed confocal z-stacks showing vehicle-treated CVP trenches compared to DMSO-treated organoids immunostained for PLCβ2 (green) and GUST (magenta). In the CVP, coarse dashed lines delineate basement membrane and apical surface of epithelium, fine dashed lines encircle individual taste buds. Unpaired t-test. Mean +/- SEM (** $p \leq 0.01$).

Development • Supplementary information

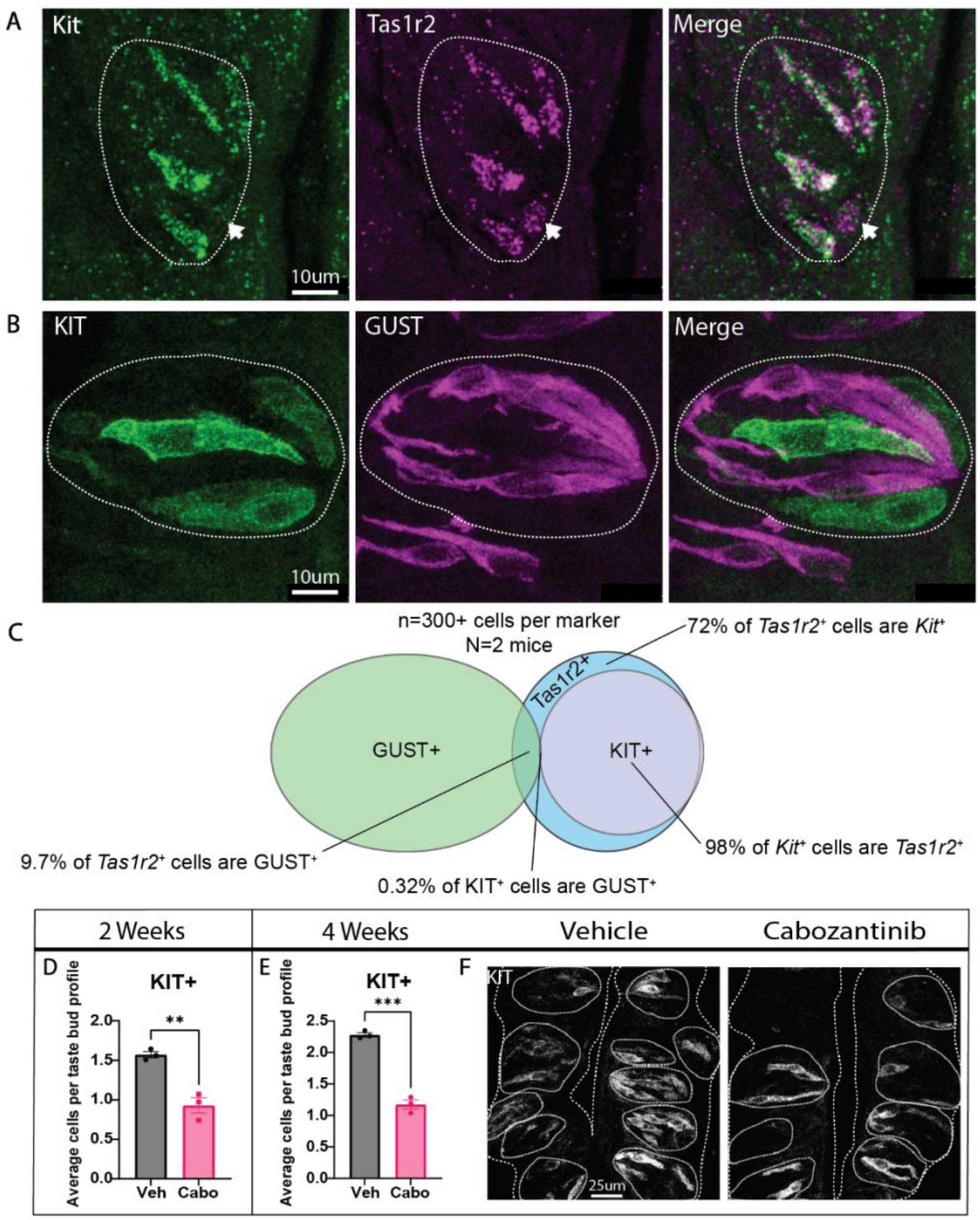

Fig. S6. *Kit* marks *Tas1r2*$^{+}$ sweet cells in the CVP. (**A**) In the CVP, HCR *in-situ* hybridization for *Kit* and *Tas1r2* reveals extensive but not complete co-expression. Arrowhead: *Tas1r2*$^{+}$/*Kit*^{neg} cell. (**B**) KIT and GUST protein expression do not overlap in CVP taste buds. Dashed lines circle individual taste buds in A and B. (**C**) Venn diagram of expression reveals all *Kit*$^{+}$ cells are *Tas1r2*$^{+}$, while GUST^{+} cells are all KIT^{neg}. (**D-F**) KIT^{+} taste cells per taste bud profile are reduced in the CVP of mice treated with cabozantinib compared to controls at 2 (**D**) and 4 weeks (**E**). (**F**) Compressed confocal z-stacks of KIT immunostaining in mice treated with vehicle or cabozantinib after 4 weeks. Coarse dashed lines delineate basement membrane and apical surface of epithelium; fine dashed lines encircle individual taste buds (N=3 mice per condition). Unpaired t-test. Mean+/-SEM (** p≤0.01, **** p≤0.0001).

Development • Supplementary information

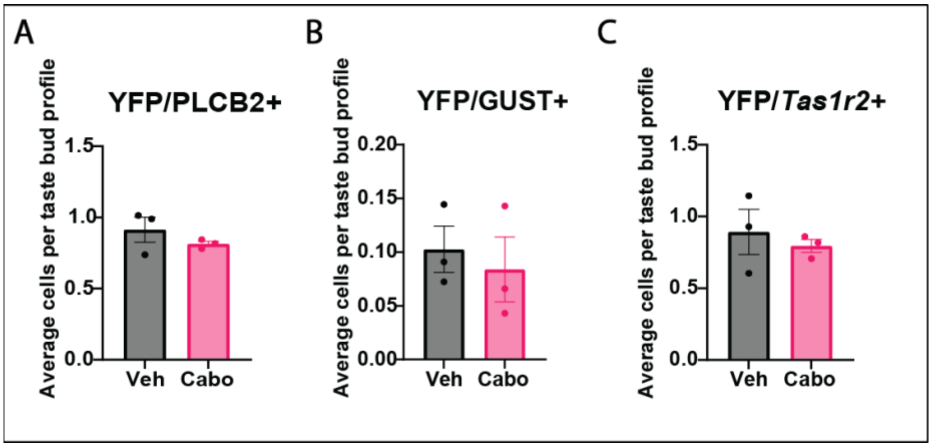

Fig. S7. Cabozantinib does not affect the average number of *Kit*-YFP⁺ cells co-expressing type II cell markers in the CVP. The average number of *Kit*-YFP⁺ lineage-traced cells expressing PLCβ2 (**A**), GUST (**B**), and *Tas1r2* (**C**) was unaltered by cabozantinib treatment.

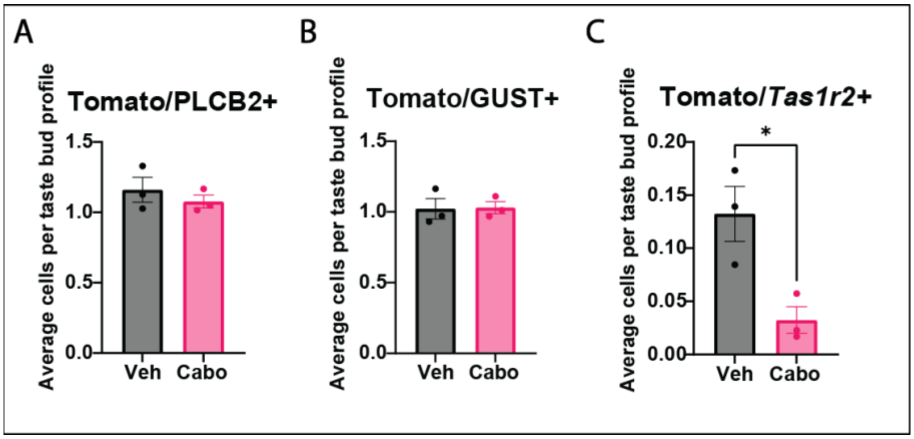

Fig. S8. Cabozantinib prevents differentiation of sweet cells in the CVP. The average number of Shh-Tomato+ lineage-traced cells expressing PLCB2 (**A**) and GUST (**B**) were not affected, while the average number of Shh-Tomato+ cells expressing *Tas1r2* (**C**) significantly decreased with cabozantinib treatment..

Development • Supplementary information

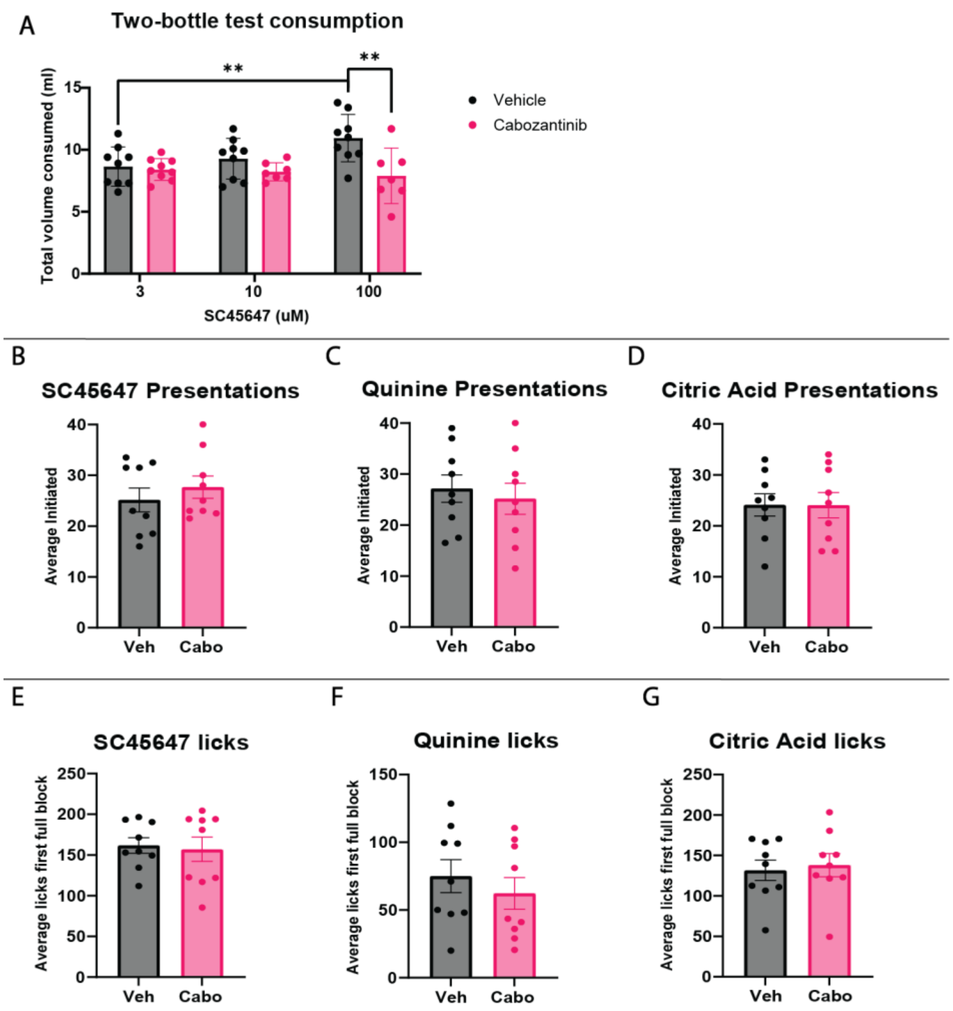

Fig. S9. Cabozantinib does not change participation effort or thirst of mice in taste behavior assays. (**A**) Total consumption of liquid across 48 hrs of testing at each concentration of tastant did not differ between control and TKI-treated mice, except at the highest concentration of SC45647 where control mice drank significantly more than drug treated mice. (**B-D**) Average presentations initiated across 2 days of lickometer testing for SC45647 (**B**), quinine (**C**), and citric acid (**D**) did not differ with treatment. The maximum number of presentations per testing session was 40. (**E-G**) Average number of licks during the first full testing block (one presentation of each concentration) across 2 testing days did not differ for SC45647 (**E**), quinine (**F**), and citric acid (**G**). Two-way ANOVA with both Sidak's and Tukey's multiple comparisons tests performed on data in panel A (N = control 9 and 7 TKI mice). Unpaired t-tests performed on panels B-G (N=9 mice per condition). Mean+/-SEM (** $p \leq 0.01$).

Development • Supplementary information

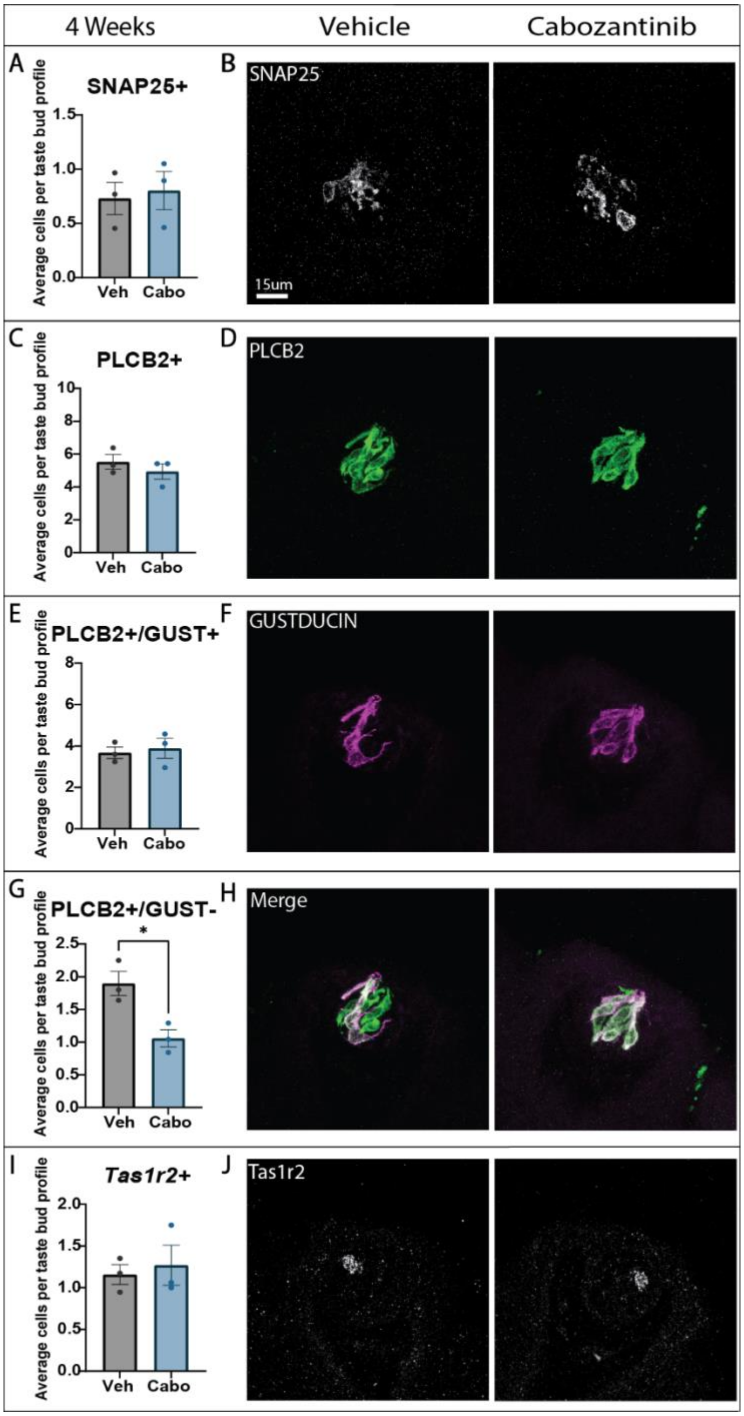

Fig. S10. Cabozantinib changes the composition of PLCβ2+ type II subtypes in FFP taste buds. Quantification of the average number of taste cells per taste bud profile with corresponding representative images for SNAP25-IF (**A-B**), PLCβ2-IF (**C-D**), PLCβ2^{+}/GUST^{+} IF (**E-F**), PLCβ2^{+}/GUST^{-} IF (**G-H**) and *Tas1r2* HCR *in situ* hybridization (**I-J**). Representative images are compressed confocal z-stack projections. Scale bar in B applies to D, F, H and J. In all histograms, each dot represents the average taste cell tally from one mouse (N=3 per condition, ~10-30 taste buds/mouse). Unpaired t-test. Mean+/-SEM (* p≤0.05).

Development • Supplementary information

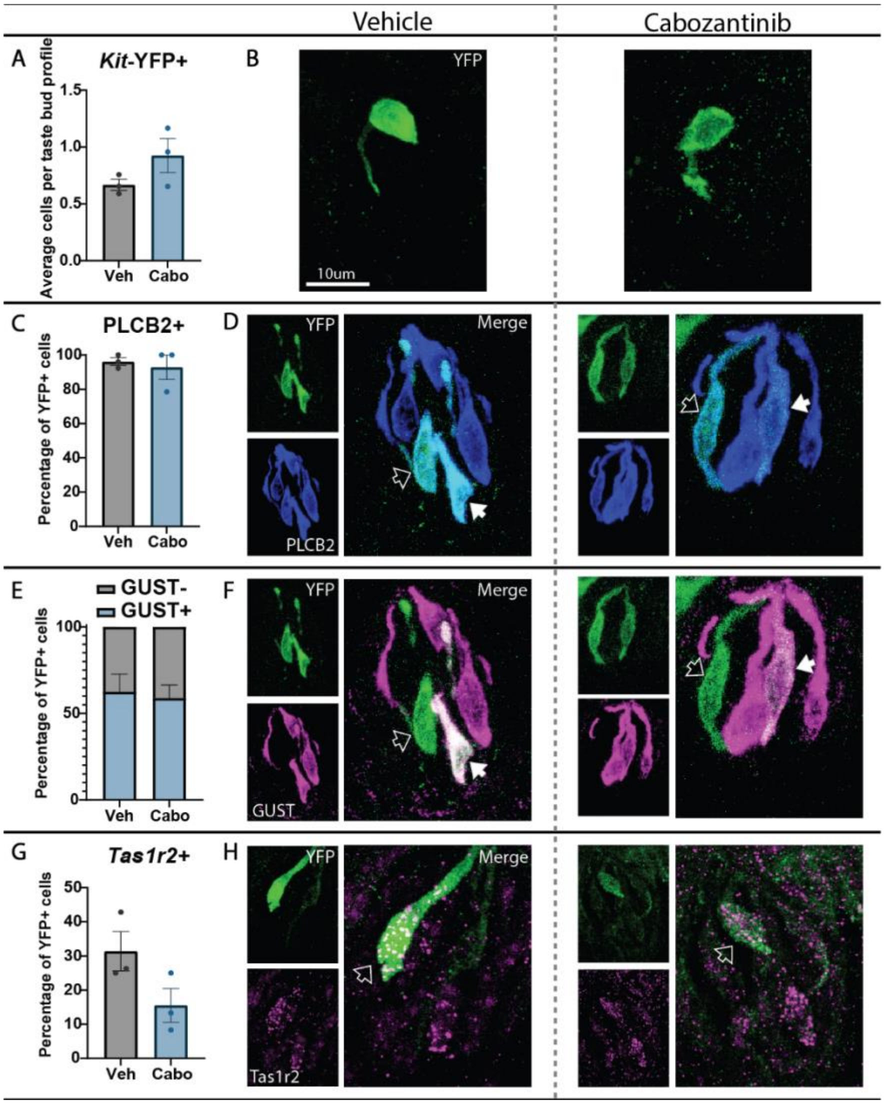

Fig. S11. Cabozantinib does not induce cell death or transdifferentiation of *Kit*$^{+}$ cells in FFP taste buds. (**A**) In *Kit*$^{CreER/+}$*;Rosa26*$^{YFP/YFP}$ mice, the average number of *Kit*-YFP^{+} cells per taste bud profile is unaltered by TKI treatment. (**B**) Compressed confocal z-stacks of *Kit*-YFP^{+} taste cells in FFP taste buds from vehicle- vs cabozantinib-treated mice. (**C, E, G**) The percentage of *Kit*-YFP^{+} cells (green in all panels) co-expressing PLCβ2 (**C-D, blue in D**), GUST (**E-F, Magenta in F**) or *Tas1r2* (**G-H, magenta in H**) is unchanged by drug treatment (compressed confocal z-stacks in all panels). Empty arrowheads in **D**, **F** and **H** indicate double-labeled cells, white arrowheads in **D** and **F** indicate triple-labeled cells. In all panels, values were calculated across >50 YFP^{+} cells and >30 taste buds per condition. Unpaired t-test performed for all quantifications. Mean+/-SEM.

Development • Supplementary information

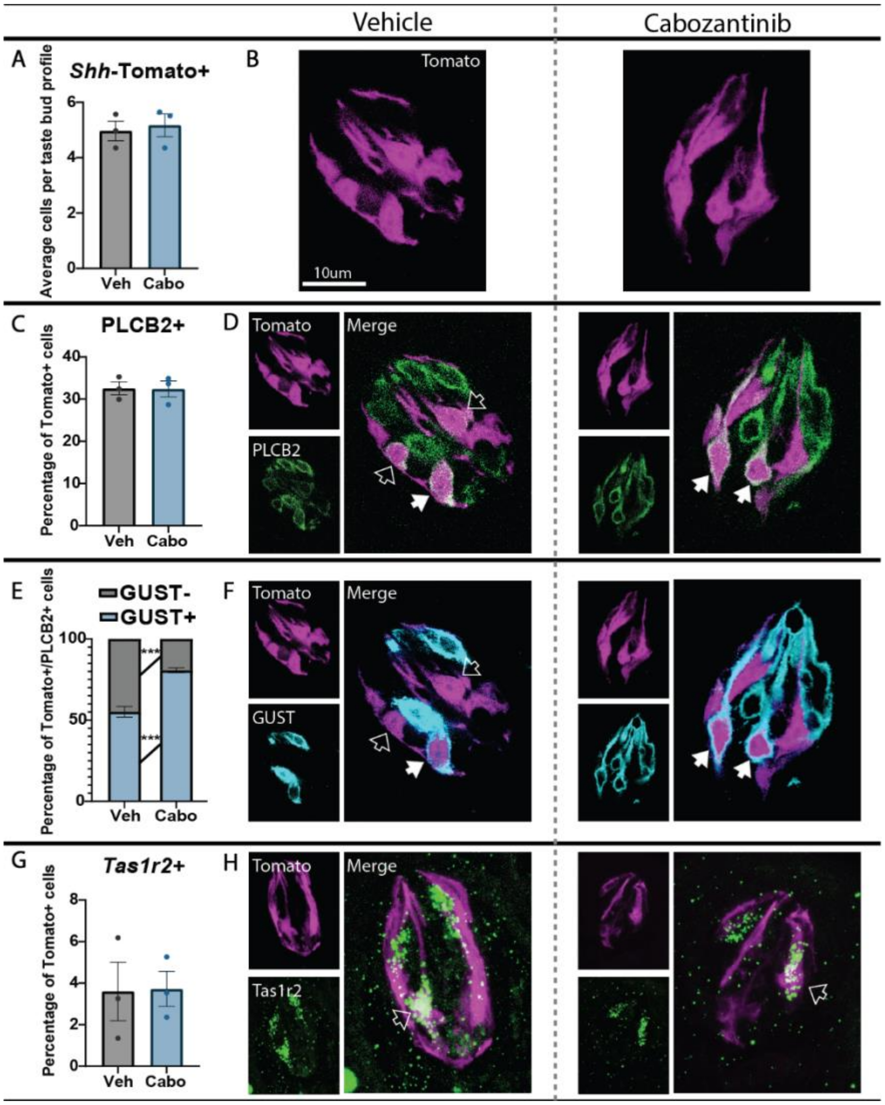

Fig. S12: Cabozantinib increases differentiation of PLCß2^{+}/GUST^{+} cells in FFP taste buds. (**A**) In *Shh*$^{CreER/+}$*;Rosa26*$^{tdTomato/+}$ mice, the average number of *Shh*-Tomato^{+} cells per taste bud profile was unaltered by cabozantinib treatment. (**B**) Optical section of *Shh*-tomato^{+} taste cells in FFP taste buds from vehicle- vs cabozantinib-treated mice. (**C, E, G**) The percentage of *Shh*-Tomato^{+} cells (**magenta** in all panels) co-expressing PLCβ2 did not change (**C-D, green in D,** optical section). The percentage of *Shh*-Tomato^{+} cells expressing GUST significantly increased and the percentage not expressing GUST significantly decreased (**E-F, cyan in F,** optical section), while the percentage of *Shh*-Tomato^{+} expressing *Tas1r2* did not change (**H-I, green in I,** compressed z-stacks). Empty arrowheads in **D**, **F** and **H** indicate double-labeled cells, white arrowheads in **D** and **F** indicate triple-labeled cells. In all panels, values were calculated across >550 Tomato^{+} cells and >95 taste buds per condition. Unpaired t-test performed for panels**A**, **C** and **G**. Two-way ANOVA with Sidak's multiple comparisons test performed for panel **E**. Mean +/-SEM (*** p≤0.001).

Development • Supplementary information

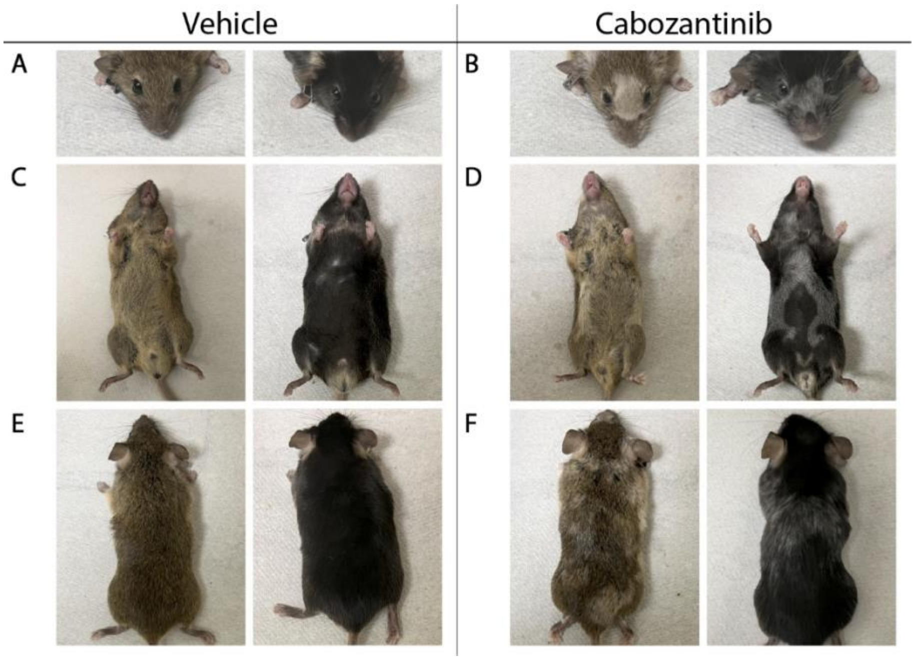

Fig. S13. Cabozantinib treatment causes fur depigmentation. Cabozantinib causes depigmentation of facial (**A-B**), stomach (**C-D**) and back (**E-F**) fur. Depigmentation was evident in mice of mixed background with brown or black fur. Images after 4 weeks of dosing.

Development • Supplementary information

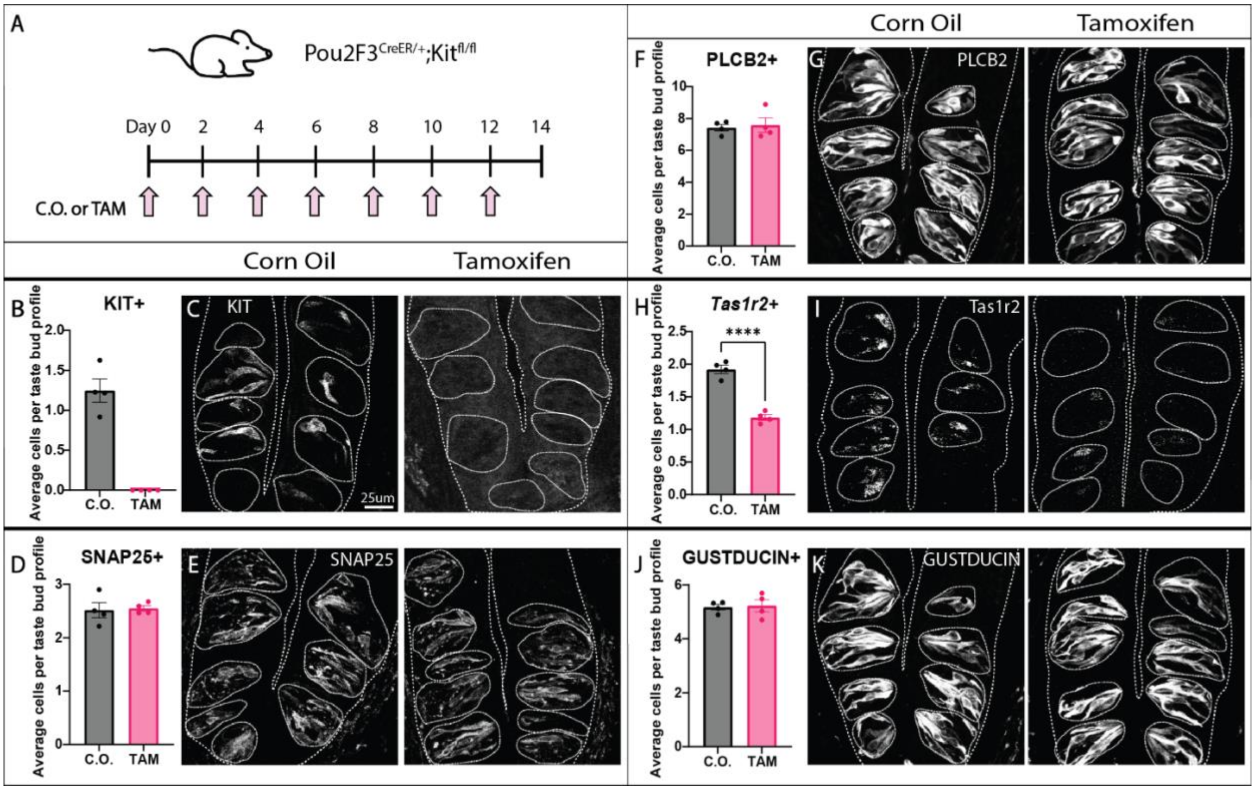

Fig. S14. Two-week conditional knockout of *Kit* reduces sweet cells but does not affect bitter/umami cells in CVP taste buds. (**A**) *Pou2F3*$^{CreER/+}$*;Kit*$^{fl/fl}$ mice were gavaged with corn oil or tamoxifen every other day for 14 days. (**B-K**) Quantification of stained cells per taste bud profile with corresponding representative images for KIT-IF (**B-C**), SNAP25-IF (**D-E**), PLCβ2-IF (**F-G**), *Tas1r2* HCR *in situ* hybridization (**H-I**) and GUST-IF (**J-K**). Representative images are compressed confocal z-stacks. Coarse dashed lines delineate basement membrane and apical epithelial surface; fine dashed lines encircle individual taste buds. Scale bar in **C** applies to **E**, **G**, **I** and **K**. In all histograms, each dot represents the average TBC tally from each mouse (N=4 per condition, ~80 taste buds/mouse). Unpaired t-test performed on all quantifications. Mean+/-SEM (**** $p \leq 0.0001$).

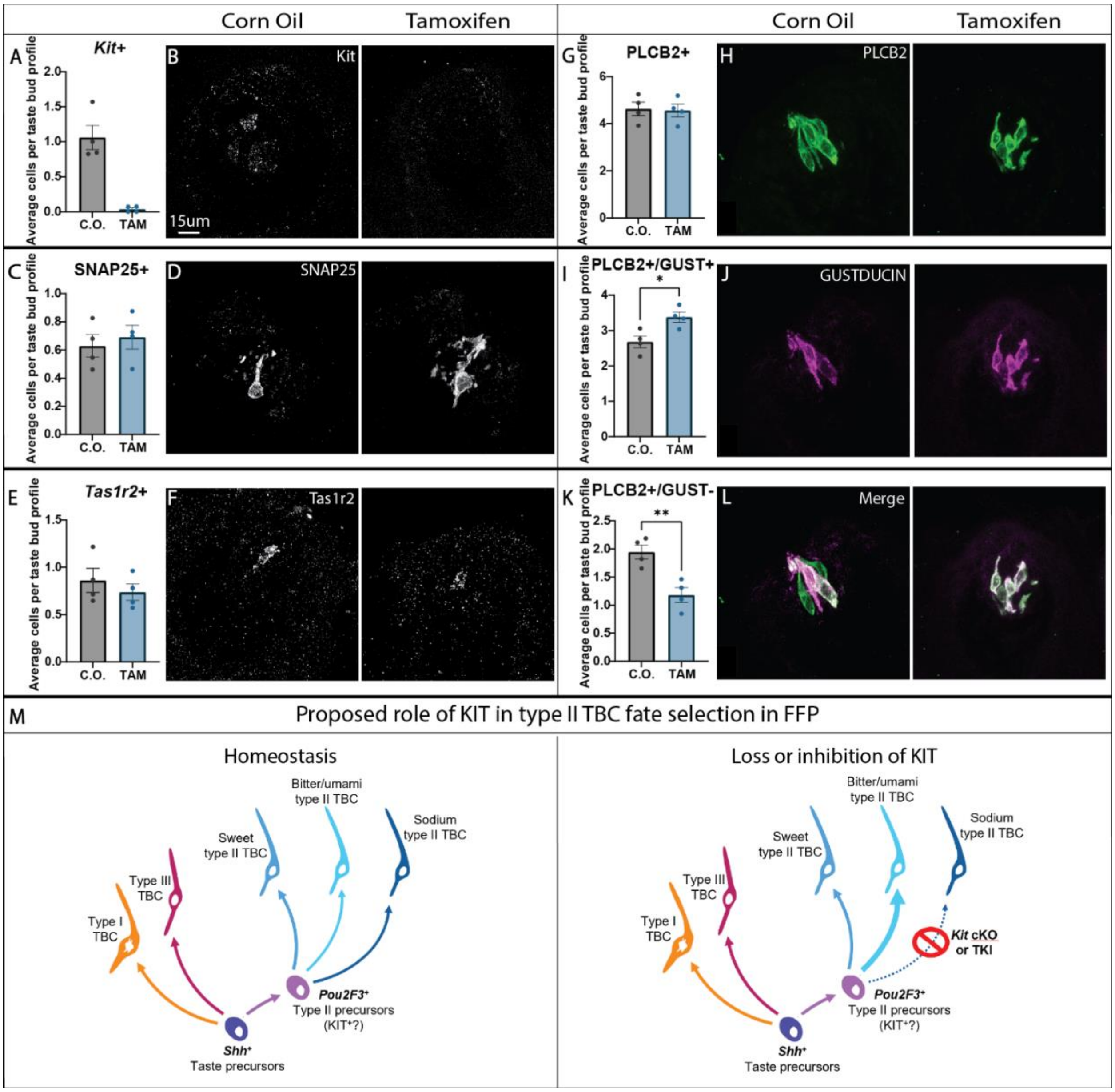

Fig. S15. ***Kit*** **knockout causes a fate switch in PLCβ2+ cells in FFP taste buds.** (**A-L**) Quantification of the average number of stained cells per taste bud profile with corresponding representative images for *Kit* HCR *in situ* hybridization (**A-B**), SNAP25 IF (**C-D**), *Tas1r2* HCR *in situ* hybridization (**E-F**), PLCβ2 IF (**G-H**), PLCβ2^{+}/GUST^{+} IF (**I-J**), and PLCβ2^{+}/GUST^{-} IF (**K-L**). Representative images are compressed confocal z-stack projections. Scale bar in B applies to D, F, H, J and L. In all histograms, each dot represents the average taste cell tally from one mouse (N=4 per condition, ~15 taste buds/mouse). Mann-Whitney test performed on data from panel A, unpaired t-test performed on data from panels C, E, G, I, K and M. Mean +/- SEM (* p≤0.05, ** p≤0.01). (**M**) Proposed model of *Kit* function in type II TBC lineage. We propose that a *Pou2f3*$^{+}$ precursor population gives rise to all type II TBC subtypes. Our pharmacological and genetic data support a model where KIT function is required for sodium-sensing type II cell fate in FFP.

Development • Supplementary information

Table S1. RT-qPCR Primers

Gene name	Refseq	Forward Sequence (5' --> 3')	Reverse Sequence (5' --> 3')
Rpl19	NM_009078.2	GGTCTGGTTGGATCCCAATG	CCCGGGAATGGACAGTCA
Kcnq1	NM_008434.2	TTTGTTCATCCCCATCTCAG	GTTGCTGGGTAGGAAGAG
Krt13	NM_010662.2	TCATCTCGGTTTGTCACTGGA	TGATCTTCTCGTTGCCAGAGAG
Kcnj1	NM_001168354.1	GACAGCTGAATGGGTGAGGT	ATCAGATGCCCTGAAACTGG
Pkd2l1	NM_181422	TACAGCGACCCTCCTTCCC	CCTCTGATGCTCCGACAGATATG
Plcb2	NM_177568.2	GAGCAAATCGCCAAGATGAT	CCTTGTCTGTGGTGACCTTG
Gnat3	NM_001081143.1	ATCCAGGAATCCAAGCCTGC	TGGTTTTCACCCGGGAATGT
Tas1r2	NM_031873.1	TGGCAGCTACTCAGGGAGAT	GAGTAGGAGGCGATGCTTTG
Pcdh20	NM_178685	GGGGCAAGCATCAAAACACATT	AAACACCAAGCAAGCAAGTAGAGATT

Table S2. Primary and secondary antibodies

**Primary Antibody**	**Source**	**Dilution**
Rat anti KRT8	DHSB; Troma-IS<br>RRID: AB_531826	1:250
Rabbit anti NTPDASE2	CHUQ; mN2-36LI6	1:300
Guinea Pig anti PLCβ2 (tissue)	Phosphosolutions; Purple antiserum<br>Bleed: 8/24/21<br>RRID: AB_3097715	1:500
Guinea Pig anti PLCβ2 (organoids)	Phosphosolutions; Blue affinity purified<br>Bleed: 8/24/21<br>RRID: AB_2934326	1:250
Goat anti GUSTDUCIN	Aviva Systems Biology; OAEB00418<br>RRID: AB_10882823	1:1,000 (tissue), 1:500 (organoids)
Goat anti SNAP25	GeneTex; GTX89577<br>RRID: AB_10724125	1:200
Goat anti CAR4	R&D Systems; AF2414<br>RRID: AB_2070332	1:50
Rabbit anti KIT	Cell Signaling Technology; 3074<br>RRID: AB_1147633	1:200
Chicken anti GFP	Aves Labs; GFP 1020<br>RRID: AB_10000240	1:500
**Secondary Antibody**	**Source**	**Dilution**
Donkey anti Goat 488	Invitrogen; A11055<br>RRID: AB_2534102	1:1,000
Donkey anti Goat 546	Molecular Probes; A11056<br>RRID: AB_2534103	
Donkey anti Guinea Pig 488	Jackson ImmunoResearch; 706-545-148<br>RRID: AB_2340472	
Donkey anti Guinea Pig 647	Jackson ImmunoResearch; 706-605-148<br>RRID: AB_2340476	
Donkey anti Chicken 488	Jackson ImmunoResearch; 703-545-155<br>RRID: AB_2340375	

Development • Supplementary information

Donkey anti Rabbit 488	Invitrogen; A21206<br>RRID: AB_2535792	
Donkey anti Rabbit 546	Invitrogen; A10040<br>RRID: AB_2534016	
Donkey anti Rabbit 647	Molecular Probes; A31573<br>RRID: AB_2536183	
Donkey anti Rat 647	Jackson ImmunoResearch; 712-605-150<br>RRID: AB_2340693	
